# Pancreatic intraepithelial neoplasia with carcinoma in situ with repeated distally localized pancreatitis: a case report

**DOI:** 10.1186/s40792-022-01369-x

**Published:** 2022-01-22

**Authors:** Yoshiaki Tanji, Kenei Furukawa, Yoshihiro Shirai, Koichiro Haruki, Shinji Onda, Takeshi Gocho, Toru Ikegami

**Affiliations:** Division of Hepatobiliary and Pancreas Surgery, Department of Surgery, The Jikei University School of Medicine, 3-25-8, Nishi-Shinbashi, Minato-ku, Tokyo, 105-8461 Japan

**Keywords:** Acute pancreatitis, Pancreatic cancer, Pancreatic intraepithelial neoplasia, Pancreatectomy

## Abstract

**Background:**

Acute pancreatitis could be an early symptom of pancreatic cancer. However, repeated pancreatitis caused by pancreatic cancer is very rare.

**Case presentation:**

A 69-year-old man was referred to our hospital with severe abdominal pain, and serial imaging studies showed acute distally localized pancreatitis with a pseudocyst. Although he had successful conservative medical treatment followed by discharge from the hospital, he was re-admitted with severe abdominal pain for recurrent distal pancreatitis with splenic artery aneurysm followed by its rupture. No pancreas mass was detected by imaging studies including endoscopic ultrasound and cytologic studies of the pancreas juice did not show any malignant cells, although slight dilatation of distal pancreas duct was observed only in the initial computed tomography. Because of the episodes of repeated distally localized pancreatitis caused by possible pancreatic ductal neoplasm, we planned and performed laparoscopy-assisted distal pancreatectomy after full-informed consent. Pathological examination revealed pancreatic intraepithelial neoplasia (PanIN) with carcinoma in situ in the distal main pancreas duct. The post-surgical course of the patient was uneventful and he was discharged 10 days after surgery from recurrent disease for over a year.

**Conclusions:**

We encountered a case of repeated episodes of acute distally localized pancreatitis, for which distal pancreatectomy was performed, resulting in pathological diagnosis of PanIN with carcinoma in situ.

## Background

Pancreatic cancer has been recognized as fatal disease process with a poor prognosis. Its incidence has been increasing in recent years. The 5-year survival rate in the United States is reported to be less than 15% [1]. Surgical resection is the only curative treatment; however, only approximately 20% of pancreatic cancers can be resected at the time of diagnosis due to the advanced stage of the cancer, as early diagnosis is challenging in pancreatic cancer cases [2].

Acute pancreatitis could be an early symptom of pancreatic cancer, and chronic pancreatitis is a risk factor of pancreatic cancer [3]. Therefore, a thorough investigation of these diseases may assist in the early diagnosis of pancreatic cancer. Here, we report a case of repeated episodes of acute distally localized pancreatitis, for which distal pancreatectomy was performed, resulting in pathological diagnosis of pancreatic intraepithelial neoplasia (PanIN) with carcinoma in situ.

## Case presentation

**Fig. 1:**
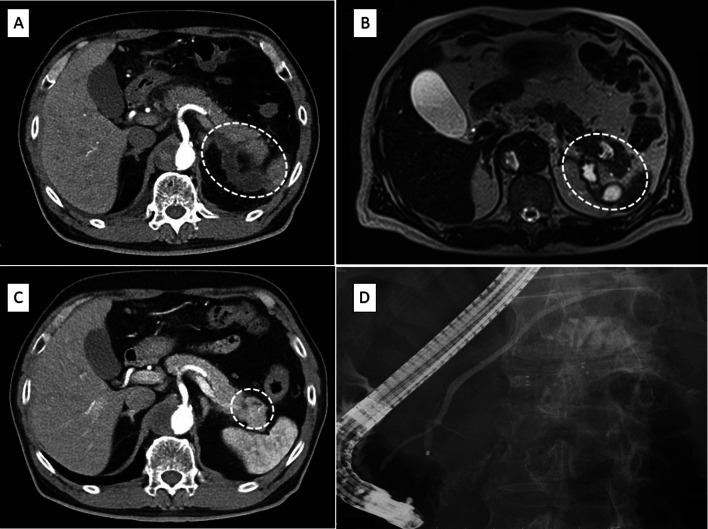
**A** Contrast-enhanced computed tomography showing fluid collection around the pancreas with pseudocyst (circle). **B** Magnetic resonance cholangiopancreatography showing pseudocyst without intraductal papillary mucinous neoplasm (circle). **C** Contrast-enhanced computed tomography showing shrinkage of the pseudocyst with slightly dilated distal pancreas duct (circle). **D** Endoscopic retrograde cholangiopancreatography showing no stenosis and dilatation of the pancreatic duct

**Fig. 2:**
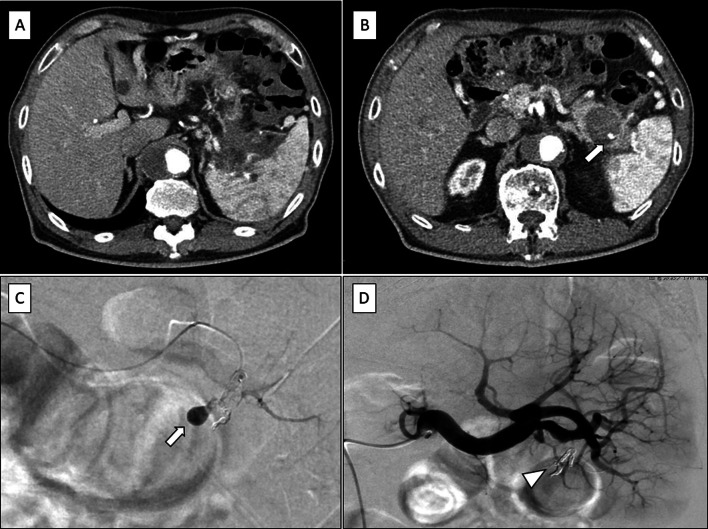
**A** Contrast-enhanced computed tomography showing fluid collection around the pancreatic tail. **B** With pseudoaneurysm of the splenic artery (arrow). **C** Abdominal angiography showing pseudoaneurysm of the splenic artery (arrow). **D** Transcatheter arterial embolization was performed (arrowhead)

A 69-year-old man visited our hospital with a complaint of acute onset abdominal pain 3 years previously. Laboratory investigation revealed that his serum amylase level was high, and computed tomography (CT) revealed fluid collection around the pancreas and a pseudocyst (Fig. 1A). He was diagnosed with acute pancreatitis without gallstones. He had no history of diabetes or any other medical conditions. The smoking history was 20 cigarettes/day for about 50 years, and the drinking history was about 500 ml of beer about 2 times a week. Magnetic resonance cholangiopancreatography (MRCP) revealed a pseudocyst without an intraductal papillary mucinous neoplasm (IPMN) (Fig. 1B). The patient was discharged, and CT recorded 1 year after discharge revealed shrinkage of the pseudocyst with slightly dilated distal pancreas duct (Fig. 1C). He developed acute pancreatitis 1 year previously. Subsequently, acute pancreatitis recurred three times. Endoscopic retrograde cholangiopancreatography (ERCP) revealed no stenosis or dilatation of the pancreatic duct at that time (Fig. 1D), and repeated pancreatic juice cytology showed no evidence of malignancy. Endoscopic ultrasound (EUS) was performed; however, no mass lesion was found in the distal pancreatic parenchyma. Acute pancreatitis recurred again this year, and CT revealed fluid retention around the tail of the pancreas and the splenic hilum, and a pseudoaneurysm of the splenic artery (Fig. 2A, B). Therefore, abdominal angiography and transcatheter arterial embolization was performed (Fig. 2C, D).

**Fig. 3:**
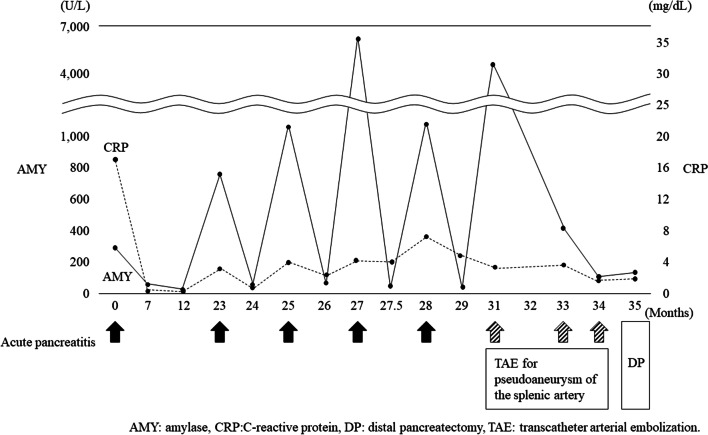
Clinical course

The possibility of pancreatic cancer could not be completely ruled out for causing repeated episodes of distally localized pancreatitis, and to treat secondary splenic artery aneurysm; therefore, surgical resection was planned after full-informed consent (Fig. 3).

**Fig. 4:**
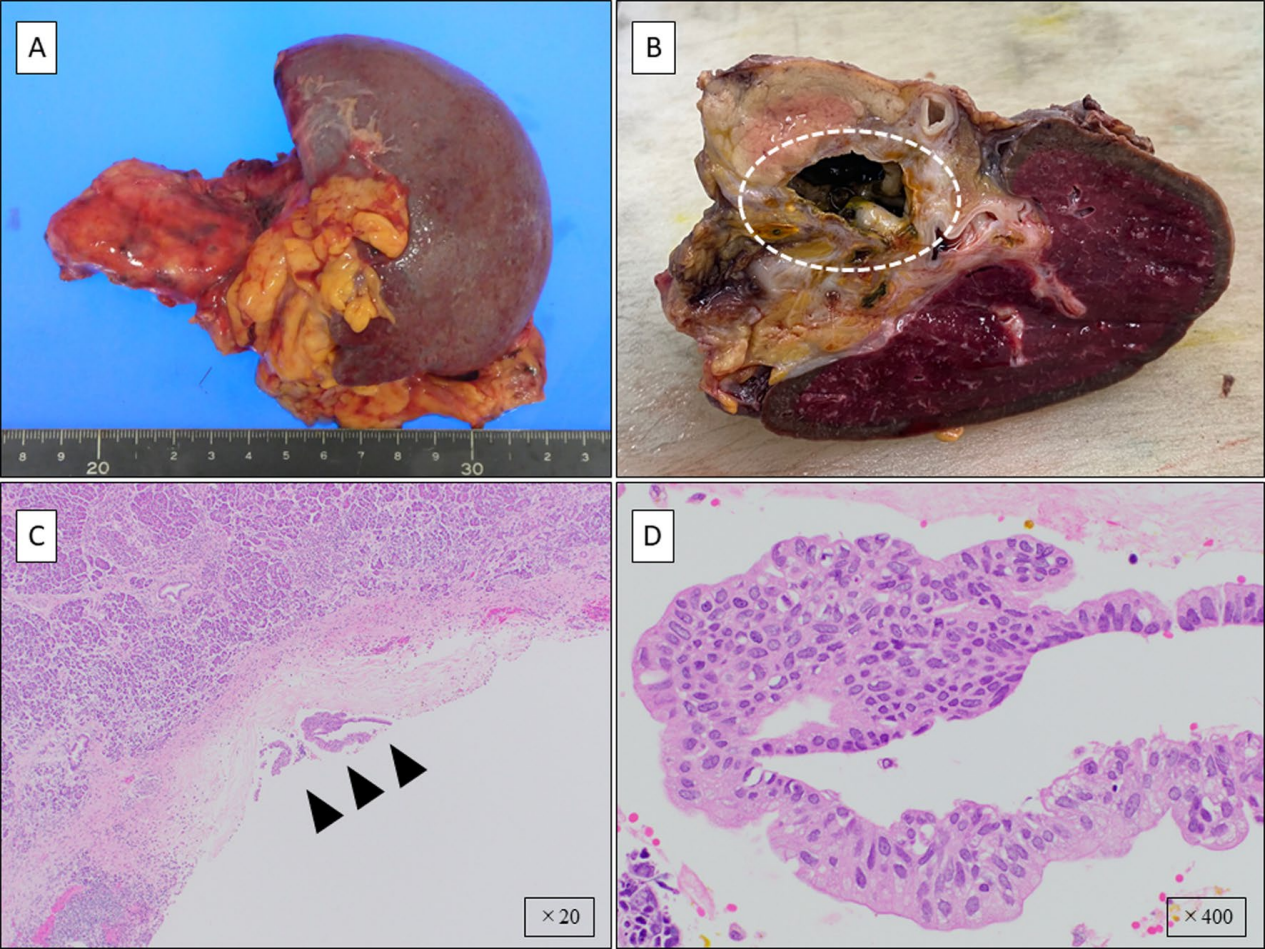
**A** Resected specimen. **B** Pancreatic pseudocyst and embolic coil exposed inside the cyst (circle). **C** Pathological diagnosis shows pancreatic intraepithelial neoplasia (black arrowhead) (×20). **D** Pathological diagnosis shows non-invasive pancreatic intraepithelial neoplasia with the strong nuclear atypia (×400)

The tumor markers 2 months prior to surgery were within the normal range as follow; carcinoembryonic antigen 4.5 ng/mL, carbohydrate antigen 19–97 U/mL, and Duke pancreatic monoclonal antigen type 2 25 U/mL. Laparoscopy-assisted distal pancreatectomy was performed, with an operation time of 293 min, and a blood loss of 200 mL (Fig. 4A). The pancreas was resected on the left side of the portal vein. Preoperative examination revealed no findings suspicious of malignancy and considering the severe inflammatory changes due to repeated pancreatitis, D1 lymph node dissection was performed without rapid intraoperative pathology of pancreatic stump. A single tufted cyst 25 mm in size was found in the tail of the pancreas and a coil embolus was found in the dilated splenic artery; a part of the blood vessel wall was ruptured, and the coil embolus was exposed in the cyst (Fig. 4B). Pathological examination revealed a non-invasive cancer component in the specimen, i.e., carcinoma in situ (Fig. 4C, D). No lymph node metastasis was detected, and PanIN with carcinoma in situ was finally diagnosed.

The patient recovered satisfactorily and was discharged on postoperative day 10. The pancreatitis did not recur after surgery and there was no recurrence of pancreatic cancer; the patient still has regular follow-ups at our hospital.

## Discussion

Surgical resection is the only curative treatment for pancreatic cancer; however, there are many cases in which resection is impossible due to delayed diagnosis, and it is reported that 15–20% of cases can be resected at the time of diagnosis [2]. Therefore, early detection of pancreatic cancer is critical. The risk factors for pancreatic cancer include a family history of pancreatic carcinoma, inherited cancer syndromes linked to pancreatic cancer, IPMN, chronic pancreatitis, high body mass index, lack of physical activity, diabetes, alcohol consumption, and cigarette smoking [4–7].

The incidence of acute pancreatitis is increasing, with a median increase of 3.4% annually [8]. Several theories have been proposed to elucidate the association between acute pancreatitis and pancreatic cancer. For instance, the risk factors for acute pancreatitis, such as alcohol and tobacco use, are similar to those for pancreatic cancer. And there are several predictors of patients who develop acute pancreatitis due to pancreatic cancer, such as age range of 56–75 years at the time of acute pancreatitis diagnosis, new onset pancreas-associated diseases [9].

The main mechanism of acute pancreatitis secondary to pancreatic cancer has been reported that the obstruction of the pancreatic duct associated with pancreatic cancer caused acute pancreatitis [3]. In this case, a pseudocyst was observed at the first onset of pancreatitis; however, no malignant mass lesions or obvious pancreatic duct disruption or dilation were detected except just after the first episode. Although the patient had recurrent episodes of distally localized acute pancreatitis thereafter, no imaging findings suggestive of malignant tumors such as mass changes and pancreatic duct changes were detected. The development of splenic artery aneurysms followed by its rupture further made the nature of distally localized pancreatitis due to severe conformational changes of his distal pancreas. In the histological diagnosis of this case, tumor cells were found in the peripheral pancreatic duct near the pseudocyst. Therefore, it was thought that repeated distally localized pancreatitis developed due to the obstruction of the peripheral pancreatic duct, because there were no pathological findings of obstructive pancreatitis around the main pancreatic duct. Although there have been two reports of repeated pancreatitis caused by tumors [10, 11], there have been no reports of such repeated localized pancreatitis as the current case.

In recent years, imaging tests such as abdominal ultrasound, CT, MRCP, and EUS have been performed for the early diagnosis of pancreatic cancer (at stages 0 and I).

The characteristic CT imaging findings in early-stage pancreatic cancer include dilation or stenosis of the main pancreatic duct and pancreatic atrophy [12]. In recent years, focal atrophy has been reported as a useful finding for carcinoma in situ in pancreatic cancer [13]. In addition, as in this case, recurrent localized acute pancreatitis may also be an important finding for the early diagnosis of pancreatic cancer.

## Conclusions

We encountered a case of repeated episodes of acute distally localized pancreatitis, where laparoscopy-assisted distal pancreatectomy was performed, and pathological examination revealed PanIN with carcinoma in situ.

## Data Availability

The data and materials of this report are able to be given from the correspond author.

## Abbreviations

CT: Computed tomography
ERCP: Endoscopic retrograde cholangiopancreatography
EUS: Endoscopic ultrasound
IPMN: Intraductal papillary mucinous neoplasm
MRCP: Magnetic resonance cholangiopancreatography
PanIN: Pancreatic intraepithelial neoplasia
TAE: Transcatheter arterial embolization
